# Song Birds as a Source of Tuberculosis

**Published:** 1899-05-27

**Authors:** 


### SONG BIRDS AS A SOURCE OF TUBERCULOSIS.

Some little time ago we referred to this subject and quoted Dr. Tucker Wise to the effect that canaries and other song birds, large numbers of which, as is well known, die of tuberculosis when kept in captivity, are a very possible cause of tuberculosis in the human subject. Dr. Wise now returns to the charge, giving a series of cases in which (although we cannot admit that his thesis is proved) there is at least sufficient evidence to raise strong suspicion that there may be some causal connection between the keeping of tuberculous song birds and the occurrence of tuberculosis among their human jailors.

It must of course not be forgotten that, so far as inoculation experiments go, avian tuberculosis is very distinct from the ordinary form as met with in cattle and human beings. The bacillus of avian tuberculosis grows at a temperature at which the human bacillus will not grow, the appearance of the growth is different, and whereas the inoculation of human tuberculosis produces in guinea-pigs a spreading disease which in the end invades most of the organs of the body, the inoculation of avian tuberculosis produces a local disease which does not spread to the internal organs. Still there is reason to believe that this difference is more of the nature of a modification resulting from altered conditions, among which the higher temperature of birds may play a considerable part, than from any essential difference in the original infection; for although on inoculating a guinea-pig with avian tuberculosis the result is but a local lesion, if this be again inoculated into a series of guinea-pigs the same typical spreading disease is produced as occurs after the inoculation of human tuberculosis. With this preface we quote the following case, related by Dr. Tucker Wise,[1] which is sufficiently striking, even by itself, to draw attention strongly to the subject. "This case occurred in a large, well-built house in one of the health resorts. The family health was good. There was no hereditary tendency to tuberculosis either in the parents or grandparents. About from twenty to thirty small birds, chiefly canaries, were kept for many years in rooms over the stable, distant about 50 ft. from the house. Many of these birds were brought into the house from time to time, and cages were always hanging in the dining-room and hall. In the kitchen a cage hung over the table on which were placed the pastry, cakes, jellies, ices, &c., for meals. In 1887 a domestic servant, aged 23 years, always delicate, developed cough with hæmoptysis. In 1889 a domestic servant, aged 22 years, became ill with cough and expectoration. In 1890 a domestic servant, aged 24 years, developed cough with hæmoptysis. In 1890 a daughter in the family, aged 30 years, well developed and enjoying good health, whose weight was 9 st. 7 lb., and who was fond of outdoor exercise, was taken ill with hæmoptysis. Caseation of the apex of the left lung supervened, and a cavity formed which had now cicatrised (1899). In 1892 a son in the family, aged 21 years, always healthy, whose weight was 11 st., and whose height was 5 ft. 10 in., was taken ill with hæmoptysis and loss of flesh, accompanied with signs of consolidation at the apex of the right lung. After two winters' treatment in the Alps no physical signs of disease remained. In 1893 a son in the family, age 27, athletic, strongly built and healthy, was taken with hæmoptysis, cough, and loss of flesh. Rapid and extensive tuberculous infiltration of the lungs supervened, and he died in 1895. This patient kept birds in his bedroom. In 1893 a domestic servant, aged 25 years, was taken ill with cough and expectoration, followed by hæmoptysis. In 1896 a daughter in the family, aged 35 years, had hæmoptysis and caseation of the apex of the left lung, with formation of a cavity. She is still under treatment." It seems clear enough that in this case some continuously infective condition was in operation for a considerable number of years. Was it the birds?

[1] Lancet, May 20.

